# Community-based trials of mobile solutions for the detection and management of cognitive decline

**DOI:** 10.1049/htl.2016.0102

**Published:** 2017-05-05

**Authors:** Adele Boyd, Jonathan Synnott, Chris Nugent, David Elliott, John Kelly

**Affiliations:** 1 The Computer Science Research Institute and the School of Computing and Mathematics, Ulster University, Newtownabbey, Co. Antrim BT37 0QB, UK; 2 Redburn Solutions Ltd., Belfast, Co. Down BT3 9DT, UK; 3 Fingerprint Learning, Bangor, Co. Down BT20 3BB, UK

**Keywords:** geriatrics, patient diagnosis, brain, cognition, health care, EnCare diagnostics, brain fit plan, cognitive assessment, colour selection, minimental state examination, early stage dementia, healthy older adults

## Abstract

This study focused on the development and usability evaluation of EnCare diagnostics (ECD) and the brain fit plan (BFP) in healthy older adults, cognitively impaired and physically impaired individuals. ECD is proposed as a novel solution to cognitive assessment based on colour selection. BFP is a novel solution to personalised cognitive stimulation. The study consisted of two trials designed to evaluate the usability of the apps. Trial 1 involved 11 healthy older adults and four older adults with physical impairments who undertook ECD and mini-mental state examination (MMSE) once per month for 4 months with only those with physical impairments also completing the BFP daily. Trial 2 involved eight older adults diagnosed with early stage dementia who completed MMSE and ECD once per month for 6 months. In Trial 1, 10 out of 11 participants enjoyed the trial and managed the usability of the app easily. A 75% drop out was observed in response to the BFP with issues of dexterity and lack of understanding on how to use the technology being the main reasons for lack of compliance. Four out of eight participants completed Trial 2 with most of the participants having no usability issues. This usability study demonstrated that ECD is highly acceptable in both healthy older adults and those with early stage dementia when given the shorter versions to accommodate their diagnosis. The BFP was not suited to this population of participants.

## 1 Introduction

The Northern Ireland Connected Health Innovation Centre (NI-CHIC) was established in June 2013 as part of an initiative to bring together Northern Ireland industry, academia and government within the field of Connected Health. Lead by Ulster University's Smart Environment Research Group and the Nanotechnology and Integrated Bioengineering Centre, this centre partners with local hardware developers, software developers, clinical organisations and care providers to develop novel technology-based interventions to assist with the monitoring and management of chronic illness. This Letter discusses the results of one study completed by NI-CHIC. This study focused on the development and usability evaluation of EnCare Diagnostics (ECD), originally designed by Redburn Solutions [1], and the Brain Fit Plan (BFP), originally designed by Fingerprint Learning [2]. ECD has been proposed as a potential alternative to the mini-mental state examination (MMSE) which is the existing gold standard for cognitive assessment. BFP has been proposed as a novel approach to personalised cognitive stimulation. The rationale behind this study originated from the literature documenting potential beneficial effects on the aging brain of domain-specific cognitive training [3, 4]. The study involved three user types: cognitively/physically healthy older adults, cognitively impaired older adults, and physically impaired older adults. ECD proposes that sensory perception of colour is objective and shared by all, and that colour preferences are subjective. ECD requires users to first select eight colours in order of preference. It then shows users a series of words and asks them to select three colours from eight colours they associate with each word – see Patent US 20140067475 A1 [5]. An education version of ECD has previously been used within the education sector to identify classroom issues with school children, and within the human resources sector [6]. This study facilitated the development of a mobile app version of ECD, in addition to a usability trial of ECD in older adults suffering from early stage dementia. The MMSE was completed alongside ECD in this usability trial (Trial 2). ECD usability was also assessed in two other cohorts: healthy older adults and older adults with physical impairments in another trial (Trial 1).

The BFP facilitates prescribed cognitive stimulation based on user preferences. Users are presented with a questionnaire which collects data about a user's learning preferences. The app then recommends a series of cognitive stimulation activities, predominantly in the form of freely available mobile apps including Chess, Sudoku, mathematics puzzles, and jigsaws, and recommends the user perform a subset of these tasks twice per day. The usability of this solution was evaluated in a trial involving older adults receiving long-term domiciliary care for physical impairment (Trial 1).

## 2 Methods and materials

The Android platform was chosen as the basis for both the ECD and BFP apps. Android was chosen due to its high market share and ease of publishing to the Google Play store. In Q2 of 2016, Android had a global market share of 87.6%. This is significantly higher than other popular mobile platforms including iOS (11.7%) and Windows Phone (0.4%) [6].

The requirements gathering process for both apps began with completing a full assessment of the existing web-based implementations of ECD and BFP, and creating a software requirements specification for each app using the IEEE format STD 830-1993. Apps recommended as the cognitive stimulation activities were selected by assessing existing apps available on the Google Play store, and assessing their usability in terms of simplicity of use, size of on-screen elements, and the presence of advertisements. BFP and ECD were developed in iterative phases, which involved regularly sharing updated versions of the apps with the project consortium as new features were implemented. This facilitated rapid feedback and amendments where necessary during the development process. Distribution of app updates was facilitated by placing the apps on the Google Play store as private beta versions which were accessible by the project consortium. Once the majority of app functionality was implemented, the apps were demonstrated to care staff from a local domiciliary care provider to receive feedback on amendments for maximising app usability.

### 2.1 Trials

The study consisted of two trials designed to evaluate the usability of the apps. Both trials were granted ethical approval by The Office for Research Ethics Committees (Northern Ireland) [Ref Nos. Trial 1 : 15/NI/0224, Trial 2 : 15/NI/0166]. All participants provided informed written consent prior to participation.

Trial 1 involved 11 healthy individuals (60 years and older with no obvious cognitive issues) recruited through the Engage with Age community development partnership and four individuals with physical impairments recruited through a local domiciliary care provider. These physical impairments would not inhibit their use of the app with colour blindness, total blindness and physical inability to hold the app being included in the exclusion criteria (Table 1). Physical impairments included a range of manifestations. These impairments required daily carer intervention to assist with activities of daily living. These physical impairments include stroke, limiting movement on one side of the body, and general mobility limitations requiring carer assistance for movement throughout the home. This trial had a duration of 4 months and involved ECD and MMSE assessment once per month, supervised by a health scientist. The participants recruited from the domiciliary care company were also asked to interact with BFP twice per day which was to be supervised by the domiciliary care staff during visits. It was envisaged that during these 15 min visits the carer would help the individual to use the BFP to complete their cognitive stimulation activities. For this reason, each of the carers recruited onto the trial underwent training on how to use the BFP and to operate the cognitive stimulation tasks offered to the participants. This occurred over two occasions and consent was taken from each carer involved in the trial to attend a focus group upon completion of the trial to gain their opinions on the burden on carer time as a result.

**Table 1:** Inclusion/exclusion criteria

Inclusion criteria	Exclusion criteria
Trial 1	
healthy older adults with expected mild cognitive impairment through aging (male or female) who may need physical help but not mental help healthy older adults over the age of 60 years access to a carer or family member who is familiar with smart phone and/or tablet technologies healthy older adults deemed psychologically fit to make responses to questions and carry out cognitive stimulation tasks and give informed consent (as assessed by the participant identification centre and also by the researcher taking consent) individuals who understand English healthy older adults who have the physical ability to hold, see and touch the mobile application	individuals <60 years of age individuals deemed psychologically unfit to make responses to questions or carry out cognitive stimulation tasks and give informed consent (as assessed by the participant identification centre and also by the researcher taking consent) individuals who do not speak or understand English individuals with a medical condition that would prevent them from being physically able to hold, see and touch the mobile application
Trial 2	
individuals with a clinical diagnosis of early stage dementia/MMSE score of around 24 (range of 21–26) (male or female) that may need physical help but not mental help individuals deemed psychologically fit to make responses to questions and give informed consent (as assessed by the participant identification centre and also by the researcher taking consent) individuals who understand English individuals who have the physical ability to hold, see (including colour blindness) and touch the mobile application	individuals with an MMSE score of lower than 21 individuals deemed psychologically unfit to make responses to questions or give informed consent (as assessed by the participant identification centre and also by the researcher taking consent) individuals who do not speak or understand English individuals with a medical condition that would prevent them from being physically able to hold, see (including colour blindness) and touch the mobile application

Trial 2 involved eight individuals diagnosed with early stage dementia as assessed using the MMSE screening tool and who fulfilled the inclusion criterion (Table 1, Trial 2) were recruited from the Alzheimer's society. This trial had duration of 6 months, involving once per month MMSE assessment and once per month ECD completion. Table 1 provides an overview of the inclusion and exclusion criteria for both trials.

Participants recruited from the domiciliary care company were provided with a study pack containing all of the necessary equipment for the trial. The key component of the pack was a Nexus 9 tablet, upon which both the ECD and BFP apps were deployed. The HTC Nexus 9 tablet was chosen due to its support for the Android platform in addition to a suitable screen size (8.90’ diagonally) for the study participants who were older adults with vision and dexterity impairments. Originally, the HTC Nexus 7 (screen size: 7.02’) was chosen; however, feedback from the staff of the domiciliary care provider suggested that this device would be too small for the end users due to vision and dexterity limitations. Participants’ user accounts were limited to only allow access to the ECD and BFP apps, removing the opportunity for accidental app removal, installation, or web browsing. Participants were also provided with a wireless SIM adaptor to provide Wifi access via 3G, and a stylus to maximise usability of the touch screen interface. Finally, a hardcopy of a user guide was provided which included images detailing the steps required to turn on the device, charge the device, log in to the user account, and open and use the apps.

### 2.2 Usability assessment

It was planned that the carer would supervise and address any problems that were experienced by the participants. Usability of the ECD was assessed during each monthly visit with each participant. This assessment involved a health scientist observing app interaction.

## 3 Results

### 3.1 Participant recruitment, follow-up, and dropout rate

In Trial 1, the recruitment rate was lower than anticipated through the privately run domiciliary care provider resulting in only *n* = 4 participants being deemed to meet the inclusion criteria. All four participants (two male and two female) completed the initial activity survey. At the beginning of the 4-month period, one participant was withdrawn from the trial due to the tasks being too onerous. Two participants passed away near the end of the 4-month test period, leaving one to complete the trial (75% dropout). The main issues reported for this initial participant were (i) The size of the tablet being too small resulting in text being too small to read. (ii) A high level of dexterity being required for some tasks such as crosswords which was not feasible. (iii) Inability to work the tablet itself through the lack of understanding. Issues that were reported by the other three participants were similar to those reported above. The issue of a high level of dexterity was partly aided by ability to use a stylus but one participant only had use of one arm due to stroke so this did not resolve the issue. One participant therefore provided four out of four sets of outcomes, one participant provided three out of four sets of outcomes, one participant provided two out of four sets of outcomes and one participant was withdrawn from the study. This participant was deemed unsuitable on attending their 2-week follow-up appointment to check progress.

Eleven healthy older adults (nine female, two male; mean age: 74 years) were recruited through the Engage with Age community development partnership to complete the ECD assessment once per month only. All of the 11 engaged with the trial and all but one completed the trial (9% dropout). The one who dropped out did so at the final time point resulting in ten full sets of follow-up data and one participant providing four out of five sets of outcomes. This participant had impaired eyesight and needed some assistance at times with reading out the words due to the text being too small. All other ten participants stated that they enjoyed the trial and managed the usability of the app easily.

Five carers who had regular visits with the trial participants agreed to be involved in the trial. It was planned that the carers would be involved in the design and development process, as well as support and encourage the participant with usage of the app. While the carers did help to inform the development of the app, on commencement of the trial, it soon became apparent that through the lack of time and inability to use the technology, there was a very low level of engagement with the participants despite the training they had received.

In trial 2, *N* = 8 participants (four female, four male, mean age: 82 years) were recruited through the Alzheimer's society. These individuals had a diagnosis of early stage dementia but no obvious physical disabilities. Four out of eight participants provided six out of six sets of outcomes, one participant provided five out of six sets of outcomes, one provided four out of six sets of outcomes, one provided three out of six sets of outcomes and one provided only two out of six sets of outcomes. Two participants were withdrawn from the study as they were deemed incapable of continued consent to continue the trial as their MMSE score had dropped below the range that was deemed acceptable to continue the study. The burden on the individual to carry out the test was also classed as being too great.

### 3.2 Usability and acceptability of brain fit plan

The majority of participants expressed dexterity limitations associated with ageing despite having no formal diagnosis of conditions affecting motor control. These dexterity limitations, combined with a lack of familiarity with touch screen technology, manifested in frequent accidental screen presses. For example, to hold the tablet comfortably, users often grasped it in such a way that their thumbs rested on the touch screen. Additionally, when using their index finger to select on-screen items, instability in movement resulted in occasionally selecting unintended items, and other areas of their hand would occasionally accidentally touch the screen. The frequency of these accidental touches was reduced through the use of a stylus, which every user preferred. In this trial, off the shelf apps were used to provide the cognitive stimulation prescribed by BFP. While every effort was taken to ensure the most suitable apps were selected, many of the apps relied on UI elements that were too small for use by those with limited dexterity and eyesight, or relied on the use of a stylus.

### 3.3 Adverse events related to brain training

Adverse events associated with this trial were limited. The only notable adverse event was occasional frustration experienced by two out of four participants. This frustration was caused by a desire to complete the brain training tasks, but occasionally struggling due to the aforementioned dexterity limitations as well as a lack of ability to work the technology.

## 4 Discussion

The main goal of Trial 1 was not to assess the effectiveness of brain training on cognitive state but to assess the usability of the approach before deployment to a larger patient population over an extended period of time, which will focus on assessment of impact. Both the carers and the participants were keen to engage with this novel technology however, results obtained from this trial suggest that this is not a feasible environment for this technology to be utilised due to the carer not having time or resources to promote participant engagement. The initial plan was to target users that receive domiciliary care twice per day and therefore engage with the technology in the same pattern; however, this was not feasible. The staffs were keen to be involved in the design and development process of both apps and provided useful feedback on the appropriateness of the apps for their client population, but did not supervise usage of BFP during each visit. This was due to time limitations, workload, and the fact one client would receive visits from many different care staff. The lack of ability by the participants to use the tablet and therefore reduce engagement may have been resolved should the participants carer have been more involved. For this particular population, bigger tablets, with easier tasks or user friendly versions would be better suited. There is an opportunity for future studies to investigate the use of ‘All in one’ touch screen computers which offer several characteristics that may mitigate the usability issues found within this trial. For example, such devices are supplied with larger displays, catering for larger on-screen user interface components and therefore minimising the impact of smaller inaccuracies in physical interaction. Additionally, these devices are typically provided with a stand which allows the device to sit upright on a table without being held. This may reduce the number of accidental interactions and would maximise usability by those with dexterity limitations.

We suggest looking further into the performance metrics of the cognitive stimulation tasks to gain a better understanding of cognitive ability and therefore possibly identify population groups who would benefit most from this type of technology. The BFP metrics were not analysed for the purpose of this Letter due to compliance being as low as most users struggled with the technology. However, most were keen to take part and expressed an interest in using an adapted version of the technology to better suit their physical needs.

The main goal of Trial 2 was to assess the usability of the ECD software and its possible validity to assess cognitive state in comparison to the widely used MMSE. Should this tool be readily accepted by such populations, it could be used to assess the effectiveness of various interventions on cognitive state. Some of the early stage dementia participants, with a lower MMSE score, struggled to complete the ECD assessment without some help. This added on significant amounts of time to the assessment visit. It soon became apparent that a shorter test would resolve this issue. A degree of subjectivity was introduced from the outcome assessor from the necessity to make a judgement on which version was applicable for the participant. Three versions were developed depending on the researcher's judgment. This resulted in those participants who were struggling to complete the trial. This subjectivity could be lessened with the production of guidelines based on the individuals MMSE score in this particular population group. All other feedback from both groups of participants was positive.

## 5 Conclusion

The ECD software proved to be a feasible and readily accepted tool to assess cognitive state in the population groups investigated, namely healthy older adults and also those individuals with early stage dementia due to the development of three versions to suit the individual being assessed.

The preliminary BFP trial would suggest that this technology as a potential service to this patient population is not feasible due to the high degree of help that is required from the carer for the participant to successfully use the technology. We recommend this technology might be more suitable in a patient population with less advanced physical and cognitive needs or individuals with a dedicated private carer who could provide the extra support needed.

Further work will investigate the potential of using larger, fixed position ‘All in one’ touch screen computers as an alternative to portable tablets, which may mitigate several of the usability issues discovered in the study.

## 6 Funding and Declaration of Interests

Invest Northern Ireland is acknowledged for supporting this project under the Competence Centre Programme Grant RD0513853 – Connected Health Innovation Centre. Dr. Elliott reports non-financial support from DAP Services a.s. during the conduct of the study.
